# Urinary bisphenol exposure and risk of type 1 diabetes and glycemic traits in Chinese children: a case–control study

**DOI:** 10.3389/fnut.2026.1880074

**Published:** 2026-07-29

**Authors:** Min Yang, Xinying Zhao, Yijie Wang, Shi Tang, Ying Xin, Jiarong Song, Yangfan Zhou, Zhongyi Liu, Lingling Zhai, Lihong Jia

**Affiliations:** 1 Department of Pediatrics, Shengjing Hospital of China Medical University, Shenyang, China; 2 Department of Maternal, Child and Adolescent Health, School of Public Health, China Medical University, Shenyang North New Area, Shenyang, China; 3 Key Laboratory of Environmental Stress and Chronic Disease Control and Prevention, Ministry of Education, China Medical University, Shenyang, Liaoning, China

**Keywords:** bisphenol A, bisphenol F, bisphenol S, glucose metabolism, type 1 diabetes mellitus

## Abstract

**Background:**

Bisphenol A (BPA) and its structural analogs, bisphenol F (BPF) and bisphenol S (BPS), are widely used in consumer products and increasingly detected in children. Although BPA has been linked to diabetes and impaired glucose metabolism in adults, evidence for BPF and BPS, particularly in the pediatric population, remains limited and inconsistent. This study examined the associations of individual and combined bisphenol exposures with type 1 diabetes mellitus (T1DM) risk and glucose homeostasis in children.

**Methods:**

We conducted a case–control study including 122 children with T1DM and 287 controls from Shenyang, China. For biomarker analysis, a subset of 110 participants (55 cases and 55 controls) was selected. Urinary bisphenol concentrations were measured using high-performance liquid chromatography–tandem mass spectrometry. Associations with T1DM risk and glycemic outcomes were evaluated using multivariable logistic and linear regression models. Bayesian kernel machine regression (BKMR) and Restricted cubic spline (RCS) analyses were further used to explore potential nonlinear relationships and interaction effects.

**Results:**

BPA was detected in all samples, with widespread exposure to BPF and BPS. Higher urinary BPA and BPF levels were significantly associated with increased odds of T1DM. BPS was not associated with T1DM status but was consistently linked to elevated fasting glucose (*β* = 0.987, 95% CI: 0.104–1.871) and HbA1c (*β* = 0.327, 95% CI: 0.012–0.642). BKMR characterized the joint associations of bisphenol mixtures with the study outcomes, whereas RCS analyses suggested near-linear exposure–response relationships between BPS and glycemic traits. Exploratory interaction analyses suggested heterogeneity in selected associations by sex and case–control status.

**Conclusion:**

Higher urinary concentrations of BPA and BPF were associated with increased odds of T1DM in children, while BPS was linked to markers of impaired glucose regulation. These findings suggest that BPA substitutes may not be biologically inert and could be relevant to metabolic health during childhood. Further prospective and mechanistic studies are needed to better understand the biological pathways underlying these associations.

## 1 Introduction

Type 1 diabetes mellitus (T1DM), the most common form of diabetes in children and adolescents, has increased globally over recent decades (1, 2). In 2021, approximately 8.4 million individuals were living with T1DM worldwide, with nearly 500,000 new cases diagnosed. The number is projected to reach 13.5–17.4 million by 2040 (3). In China, regional variations have been observed, with a higher prevalence reported in northern provinces among children under 14 years of age (4). Although longitudinal data for the northern regions remain limited, a rapidly increasing trend has been documented in southern China, with an average annual rise of 12.0–14.2% among children and adolescents (2, 5). While T1DM is primarily an autoimmune disease in genetically susceptible individuals, environmental factors are increasingly recognized as a potential contributor to disease onset and progression (6, 7).

Among the candidate environmental factors, endocrine-disrupting chemicals (EDCs) have attracted increasing attention. Bisphenol A (BPA), a ubiquitous EDC widely used in plastics, food packaging, and consumer products, has been associated with metabolic disturbances and diabetes in humans and has been widely studied for its metabolic and immunologic effects (8–12). Experimental studies in non-obese diabetic (NOD) mice suggest that BPA exposure, particularly during early life or following maternal exposure during pregnancy, may accelerate insulitis and autoimmune diabetes development (13, 14). Proposed mechanisms include immune dysregulation and impaired *β*-cell or calcium homeostasis (13, 15, 16). However, these experimental findings provide biological plausibility rather than direct evidence in humans. The available epidemiological literature is limited and methodologically heterogeneous, consisting mainly of small cross-sectional and case–control studies. Findings have been inconsistent, with some studies reporting an association between BPA exposure and T1DM risk (9, 10), whereas others found no statistically significant association (17). These inconsistencies may be attributable to differences in study populations, exposure assessment methods, and the control of potential confounding factors. Furthermore, evidence regarding BPA exposure and glycemic characteristics in children with T1DM remains limited. Although a recent study in Egyptian children with T1DM measured urinary BPA concentrations together with HbA1c and random blood glucose levels (10), it did not evaluate the associations of BPA exposure with a comprehensive set of glycemic and *β*-cell function markers. Therefore, current epidemiological evidence remains insufficient and falls far short of establishing a causal relationship between BPA exposure and T1DM.

With increasingly stringent restrictions on BPA use, bisphenol F (BPF) and bisphenol S (BPS) have been widely introduced as BPA substitutes, leading to widespread human exposure (18–20). However, evidence regarding their potential relevance to T1DM has lagged behind their expanding use. Among BPA substitutes, BPS has received comparatively greater attention (21–23). Experimental studies in non-obese diabetic (NOD) mice suggest that BPS exposure may influence autoimmune diabetes progression and glucose homeostasis, although the direction of its effects and the underlying mechanisms may differ from those reported for BPA (21). Nevertheless, epidemiological evidence linking BPS exposure to T1DM in humans remains limited. Although several studies have examined BPS in relation to type 2 diabetes or gestational diabetes (24), these findings cannot be directly extrapolated to T1DM, which is primarily characterized by autoimmune *β*-cell destruction. Evidence on the role of BPF in T1DM is even more sparse. While *in vitro* studies suggest that BPF may disrupt IRS-1/PI3K/AKT signaling and impair insulin-mediated glucose metabolism, direct evidence linking BPF exposure to T1DM is scarce (25). In addition, the potential health effects of real-world bisphenol mixtures have not been fully characterized.

Given the widespread exposure to bisphenol analogs and the growing burden of T1DM in children, there is a need to better understand the potential role of these environmental chemicals in pediatric metabolic health. Previous studies have largely focused on BPA in relation to type 2 diabetes and other metabolic outcomes, evidence regarding BPF and BPS, particularly in relation to T1DM and glycemic traits in children, remains limited. Therefore, we conducted a case–control study among children with newly diagnosed T1DM and non-diabetic controls to examine the associations of urinary concentrations of BPA, BPF, and BPS, both individually and as a mixture, with T1DM risk and glycemic outcomes. By integrating conventional regression and mixture-modeling approaches, we aimed to provide a more comprehensive assessment of the associations between urinary bisphenol exposure, T1DM risk and glycemic traits.

## 2 Materials and methods

### 2.1 Study population

This case–control study was conducted between January and December 2023 in Shenyang, China. All pediatric patients aged 1–14 years with newly diagnosed T1DM at Shengjing Hospital of China Medical University during the study period were eligible for inclusion. T1DM diagnosis was clinically confirmed by pediatric endocrinologists according to the 2023 American Diabetes Association (ADA) criteria (26). Patients diagnosed with other forms of diabetes, such as type 2 or monogenic diabetes, were excluded.

The control group comprised 287 healthy children and adolescents recruited from two schools and a kindergarten in Shenyang. Controls were frequency-matched to cases based on age, sex and body mass index (BMI). Medical history and laboratory screening were performed to ensure the absence of diabetes or impaired glucose tolerance in the participants.

**Figure 1:**
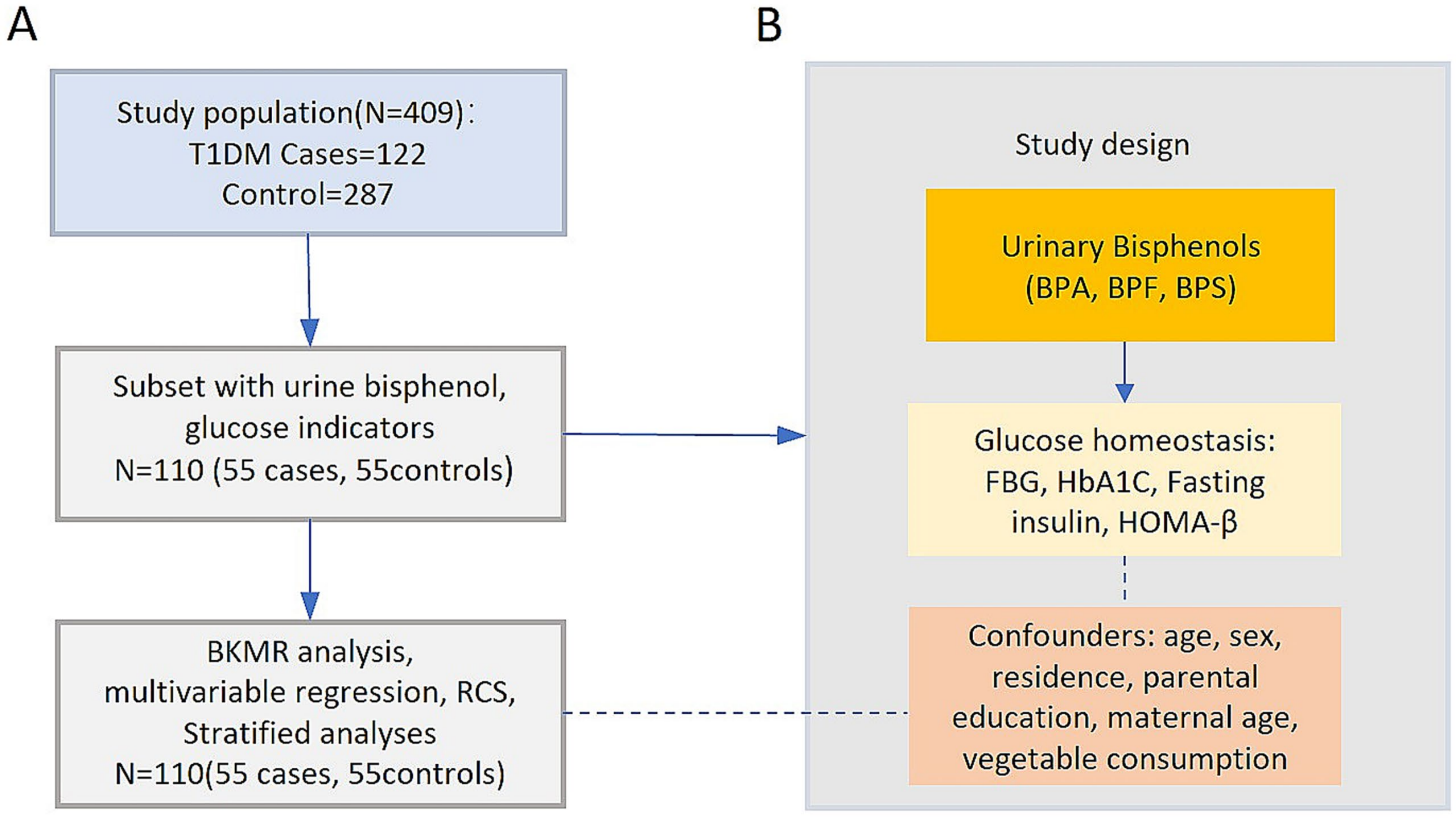
Study design and conceptual framework. **(A)** Shows the participant flow: 409 children (122 cases and 287 controls) were enrolled, and 110 (55 cases and 55 controls) were included in the biomarker analyses. **(B)** Illustrates the conceptual framework linking urinary bisphenol levels to glucose homeostasis outcomes, with adjustments for major covariates.

For bisphenol analysis, a subset of 110 children (55 T1DM cases and 55 controls) aged 6–14 years with available urine samples and complete metabolic data were included in the bisphenol analysis. These participants were selected based on specimen availability and subsequently matched by age, sex, and BMI to minimize potential confounding factors. This subsampling strategy was uniformly applied without regard to exposure levels or outcomes of interest. The distributions of age, sex, and BMI did not differ significantly between the subsamples and the full study population. The detailed study flow and participant selection processes are illustrated in Figure 1. The sample size consideration and statistical power are described in detail in Supplementary methods S1.

The study protocol was approved by the Medical Research Ethics Committee of Shengjing Hospital of China Medical University (Approval Number: 2023PS040K) and was conducted in accordance with the ethical principles of the Declaration of Helsinki. Written informed consent was obtained from the parents or legal guardians of all participating children before enrolment.

### 2.2 Questionnaire

A structured questionnaire was administered at enrollment. For children with T1DM, questionnaire information was provided by parents or legal guardians and recorded by trained healthcare staff during recruitment. For controls, questionnaires were distributed and collected through school healthcare staff and were completed by parents or legal guardians.

The questionnaire collected information on parental characteristics (parental obesity, smoking status, educational level, allergic history, maternal age at pregnancy, household income). Information on the children included birth and early-life factors, dietary habits (milk, vegetables, fruits, fast food and snacks), lifestyle factors (physical activity, screen time, sleep duration), allergic history, and general family history of diabetes. A family history of diabetes was defined as diabetes in one or more first- or second-degree relative. Missing or unclear information was verified whenever possible.

### 2.3 Assessment of bisphenols and glycemic parameters

Spot urine samples were collected on the day of hospital admission for cases and at study enrollment for controls in 50-mL polypropylene cups and stored at −20 °C until analysis. Urinary BPA, BPF, and BPS levels were quantified using pre-column derivatization coupled with ultra-high-performance liquid chromatography–tandem mass spectrometry (Agilent 1,290–6,420, USA). Standards for BPA and BPF were prepared according to previously published methods (27). ^13^C12-BPS was obtained from Toronto Research Chemicals (Toronto, Canada). The monitored ions and mass spectrometric parameters are listed in Supplementary Table S1; detailed instrument settings, Quality assurance/quality control procedures and limits of detection (LOD) and quantification (LOQ) are described in the Supplementary methods S2. Values below the limit of detection (LOD) were imputed as LOD/√2. Urinary creatinine levels were measured as previously described (27), and creatinine-adjusted (Cr-adjusted) bisphenol concentrations were expressed as μg/g creatinine (μg/g Cr). Fasting blood samples were collected after an overnight fasting ≥10 h. Serum glucose levels were determined using the hexokinase method (AutoBio kit), C-peptide and insulin levels were measured using chemiluminescent microparticle immunoassay (CMIA; Abbott GmbH & Co. KG); HbA1C assay by ion-exchange high-performance liquid chromatography (HPLC; Bio-Rad). HOMA-*β* was calculated as: 20 × fasting insulin (mU/L)/ [fasting glucose (mmol/L)-3.5].

### 2.4 Covariates

Candidate covariates included age, sex, residence, parental education, maternal age, vegetable intake, BMI, and general family history of diabetes. For analyses of glycemic traits conducted in the combined sample, case–control status was additionally included as a covariate. Detailed information on family history of T1DM and other autoimmune diseases was not available.

### 2.5 Statistical analysis

Continuous variables are summarized as medians (IQR) and categorical variables as numbers (%). Pairwise associations among urinary BPA, BPF, and BPS levels were assessed using Spearman’s correlation coefficients. The sum of the bisphenols was calculated as ΣBPs = BPA + BPF + BPS. Exposure variables were creatinine-adjusted and natural log-transformed prior to analysis.

Covariates for the primary analyses were selected *a priori* based on prior literature and causal considerations. A directed acyclic graph (DAG) was constructed to identify a minimally sufficient adjustment set for confounding control (Supplementary Figure S1). Descriptive bivariate associations between candidate covariates and study outcomes are presented in Supplementary Table S2. Primary models were adjusted for age, sex, residence, parental education, maternal age, and vegetable intake. BMI and general family history of diabetes were not included in the primary adjustment set. BMI was measured at enrollment, near the time of T1DM diagnosis, and may therefore have been affected by disease-related metabolic changes. General family history of diabetes was not retained in the DAG-derived minimally sufficient adjustment set. Sensitivity analyses further adjusted for BMI and family history of diabetes. For analyses of glycemic traits in the combined sample, models were additionally adjusted for case–control status. Multicollinearity was assessed using variance inflation factors, with all VIF values <5. Adjusted models were considered the primary analyses, whereas crude models were presented for descriptive comparison.

For T1DM (binary outcome), associations with individual bisphenols (BPA, BPF, and BPS) and their sum (ΣBPs) were estimated using multivariable logistic regression models, with results reported as odds ratios (ORs) and 95% confidence intervals (CIs). For glucose metabolism markers (fasting blood glucose (FBG), HbA1c, insulin, and HOMA-*β*), multivariable linear regression models were applied. For exploratory categorical analyses, bisphenol concentrations were categorized into tertiles (Q1–Q3), and the median value of each tertile was modeled as a continuous variable (*p* for trend).

Potential effect modification by sex (male/female) and case–control status (control/case) was assessed by adding multiplicative interaction terms between each ln-transformed urinary bisphenol concentration and the corresponding subgroup variable to the adjusted regression models. Wald *p* values for interaction were calculated. Given the limited sample size, a *p* value for interaction < 0.10 was regarded as exploratory evidence of heterogeneity. Stratified estimates were interpreted only for exposure-outcome pairs with evidence of interaction.

Mixture effects were evaluated using Bayesian kernel machine regression (BKMR) with a probit link and a Gaussian kernel. Markov chain Monte Carlo sampling was performed with 10,000 iterations, with burn-in and thinning as detailed in Supplementary methods S3. Posterior inclusion probabilities (PIPs), overall mixture effects, univariate exposure–response functions (with other exposures at specified quantiles), and bivariate response surfaces were estimated in this study.

Potential nonlinear associations between exposures and continuous outcomes were examined using restricted cubic spline (RCS) regression. Primary analyses used four knots at the 5th/35th/65th/95th percentiles, and sensitivity analyses used three knots at the 10th/50th/90th percentiles (Supplementary methods S3).

To account for multiple tests within exposure families, the Benjamini–Hochberg false discovery rate (FDR) procedure was applied with q = 0.05. Forest plots were used to visualize stratum-specific *β* estimates with 95% CIs for each outcome–exposure pair (vertical line at *β* = 0).

All statistical analyses were conducted using IBM SPSS version 25.0 (IBM Corp., Armonk, NY, USA) and R version 4.3.3 (R Foundation for packages: bkmr, rcssci, rms and ggplot2).

## 3 Results

### 3.1 Demographic characteristics of the study population

The demographic characteristics of the case and control groups are shown in Table 1. There were 122 children with T1DM and 287 healthy children who completed the questionnaire. Children with T1DM had significantly higher levels of parental education and family history of diabetes (*p* < 0.001). Children with T1DM had a larger proportion of mothers exceeding 35 years of age during pregnancy in comparison to the control cohort (*p* = 0.010). Children with T1DM seldom consumed vegetables (*p* < 0.001). Age, sex, BMI, and residence of the 110 participants who underwent bisphenol measurement are presented in the Supplementary Table S3.

**Table 1:** Characteristics of the study population.

Parameters	Cases (*n* = 122)	Controls (*n* = 287)	*x^2^/t*	*p* value
	*n* (%)	*n* (%)		
Age (years)			1.558	0.463
<5	19 (15.6)	40 (13.9)		
5–10	53 (43.4)	144 (50.2)		
≥10–14	50 (41)	103 (35.9)		
Sex			0.089	0.829
Boys	55 (45.1)	134 (46.7)		
Girls	67 (54.9)	153 (53.3)		
BMI			3.756	0.149
Normal	95 (77.9)	201 (70.0)		
Overweight	15 (12.3)	37 (12.9)		
Obesity	12 (9.8)	49 (17.1)		
Residence			83.787	<0.001
Urban	68 (55.7)	268 (93.4)		
District	13 (10.7)	7 (2.4)		
Rural	41 (33.6)	12 (4.2)		
Feeding patterns within 4 (6) months			5.797	0.053
Breast feeding	90 (73.8)	182 (63.4)		
Formula feeding	16 (13.1)	38 (13.2)		
Mixed feeding	16 (13.1)	67 (23.3)		
Father education			40.576	<0.001
Junior high school and below	54 (44.3)	43 (15.0)		
Senior high school	21 (17.2)	77 (26.8)		
College and above	47 (38.5)	167 (58.2)		
Mother education			56.831	<0.001
Junior high school and below	61 (50.0)	42 (14.6)		
Senior high school	19 (15.6)	77 (26.8)		
College and above	42 (34.4)	168 (58.5)		
Maternal age (year)			6.849	0.010
<35	101 (82.8)	263 (91.6)		
≥35	21 (17.2)	24 (8.4)		
Family history of diabetes			30.341	<0.001
Yes	59 (48.4)	61 (21.3)		
No	63 (51.6)	226 (78.7)		
Intake of snacks often			3.972	0.055
Yes	87 (71.3)	175 (61.0)		
No	35 (28.7)	112 (39.0)		
Intake of fast food often			3.633	0.071
Yes	52 (42.6)	94 (32.8)		
No	70 (57.4)	193 (67.2)		
Intake of deli meats often			3.654	0.066
Yes	67 (54.9)	128 (44.6)		
No	55 (45.1)	159 (55.4)		
Intake of fruits, times/week			0.772	0.725
<2	6 (4.9)	9 (3.1)		
2–4	19 (15.6)	45 (15.7)		
≥4	97 (79.5)	233 (81.2)		
Intake of vegetables, times/week			84.628	<0.001
<2	51 (41.8)	55 (19.2)		
2–4	63 (51.6)	73 (25.4)		
≥4	8 (6.6)	159 (55.4)		
Time spending on electronic products (hour)	1.07 (0.84,1.31)	1.04 (0.91, 1.16)	−0.757	0.450
Sleep duration (hour)	9.33 ± 2.19	9.10 ± 0.86	−1.418	0.157
Whether suffering from eczema			0.578	0.506
Yes	44 (36.1)	115 (40.1)		
No	78 (63.9)	172 (59.9)		
Whether allergies to certain foods			0.274	0.611
Yes	26 (21.3)	68 (23.7)		
No	96 (78.7)	219 (76.3)		

Values highlighted in bold are considered significant with *p* < 0.05.

### 3.2 Urinary bisphenols levels

The detection frequencies and concentrations of the creatinine-adjusted urinary bisphenols are summarized in Table 2. BPA was detected in 100% of samples; BPF and BPS were detected in 90.91 and 99.09%, respectively. The geometric mean (GM) concentrations (μg/g creatinine) were 3.95 for BPA, 1.16 for BPF, 1.51 for BPS, and 9.48 for ΣBPs. The creatinine-adjusted concentrations of BPA, BPF, and ΣBPs were significantly higher in the cases than in the controls, whereas the creatinine-adjusted BPS concentrations were lower in the cases (*p* values are shown in Table 2). Spearman correlations among bisphenols were positive, with BPA correlating with both BPF and BPS (*ρ* = 0.20–0.48; Supplementary Table S4).

**Table 2:** Urinary levels of bisphenols in T1DM and healthy children.

Concentration	>LOD	All subjects (*N* = 110)		Cases (*N* = 55)		Controls (*N* = 55)		*Z*	*p*
	Subjects (%)	GM	Median (25th-75th)	GM	Median (25th-75th)	GM	Median (25th-75th)		
Unadjusted bisphenols levels (μg/L)									
BPA	110 (100)	2.96	2.91 (1.39–6.54)	3.51	3.73 (1.43–9.56)	2.49	1.88 (1.21–4.16)	−1.952	0.051
BPF	100 (90.91)	0.87	0.86 (0.42–3.06)	1.35	1.45 (0.63–3.80)	0.56	0.58 (0.19–1.79)	−3.047	0.002
BPS	109 (99.09)	1.13	0.98 (0.56–2.82)	0.60	0.62 (0.40–0.81)	2.12	2.64 (1.49–4.35)	−7.198	<0.001
∑BPs		7.09	6.37 (4.30–12.44)	7.05	6.11 (3.80–14.33)	7.13	6.57 (4.31–9.73)	−0.093	0.929
Cr-adjusted bisphenols levels (μg/g)									
BPA	110 (100)	3.95	3.80 (1.67–8.69)	6.87	6.29 (3.48–17.39)	2.28	1.88 (1.22–4.06)	−5.126	<0.001
BPF	100 (90.91)	1.16	1.31 (0.34–4.44)	2.64	3.03 (1.39–8.32)	0.51	0.48 (0.23–1.28)	−5.192	<0.001
BPS	109 (99.09)	1.51	1.64 (0.70–3.74)	1.17	1.49 (0.45–3.09)	1.93	2.16 (0.58–5.45)	−2.125	0.020
∑BPs		9.48	8.49 (4.82–16.78)	13.80	12.70 (6.92–30.69)	6.51	6.80 (4.10–9.80)	−4.074	<0.001

GM, geometric mean; Cr, Creatinine.

### 3.3 Relationship between individual and combined exposure to bisphenols and risk of T1DM

Table 3 illustrates the associations between individual and combined urinary bisphenol exposures with the risk of T1DM. In the adjusted models, increased urinary levels of BPA (*p* < 0.001), BPF (*p* < 0.001) and ∑BPs (*p* < 0.001) were significantly correlated with an enhanced risk of T1DM. Exploratory tertile-based analyses and sensitivity analyses with additional adjustment for BMI, family history of diabetes, and both variables are presented in Supplementary Tables S5–S7.

**Table 3:** Odds ratios and 95% confidence intervals of the associations between urinary bisphenols (μg/g Cr) and T1DM.

Variable	Model 1	*p*	Model 2	*p*
	OR (95%CI)		OR (95%CI)	
BPA	2.457 (1.621, 3.723)	<0.001	2.452 (1.567, 3.837)	<0.001
BPF	2.091 (1.527, 2.863)	<0.001	2.183 (1.543, 3.087)	<0.001
BPS	0.712 (0.515, 0.984)	0.040	0.679 (0.469, 0.982)	0.040
∑BPs	2.818 (1.673, 4.747)	<0.001	2.939 (1.653, 5.225)	<0.001

Model 1 unadjusted model. Adjusted model. Model 2 was adjusted for age, sex, residence, parental education, maternal age, and vegetable consumption.

### 3.4 Joint and individual exposure of bisphenol compounds associated with the risk of T1DM employing BKMR regression models

**Figure 2:**
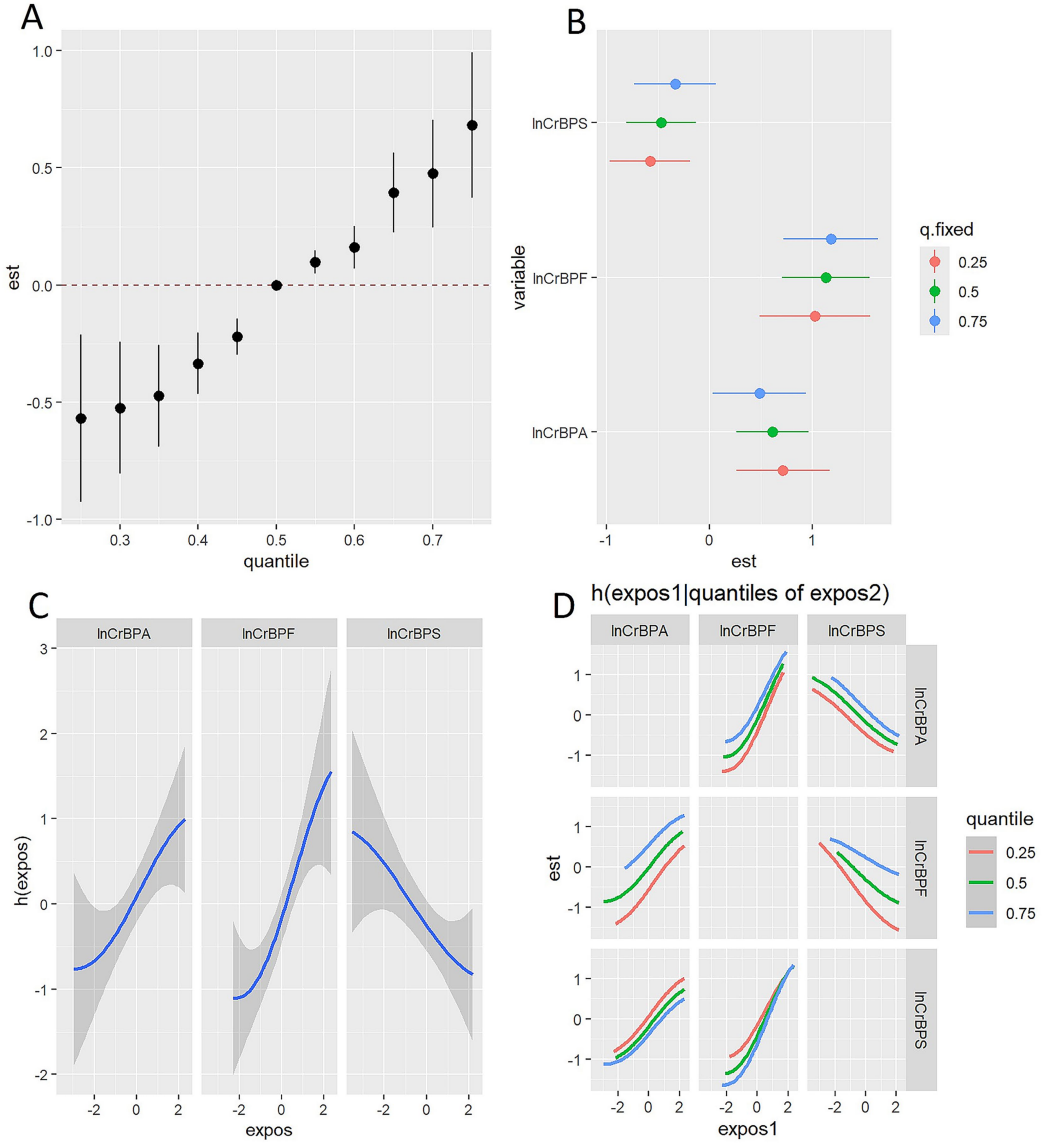
BKMR analysis of the bisphenol mixtures. The models were adjusted for age, sex, residence, case–control status, parental education, maternal age, and vegetable consumption. **(A)** Overall effect of the bisphenol mixture (lnCrBPA, lnCrBPF, and lnCrBPS) across quantiles of the joint exposure distribution on the risk of T1DM. The plot shows the estimated change in T1DM risk, with 95% credible intervals, as the mixture quantiles increase. **(B)** Single-exposure effects of each bisphenol, defined as the change from the 25th to the 75th percentile, while holding the other bisphenols at the 25th, 50th, or 75th percentiles. **(C)** Single-variable exposure-response relationships and 95% CIs (gray part) indicate the correlation between each bisphenol and T1DM risk, with other bisphenol levels tied to the median. **(D)** Pairwise bivariate exposure-response functions illustrating how the effect of one bisphenol varies across quantiles of another. The lines represent the estimated effects at the 25th (red), 50th (green), and 75th (blue) percentiles of the interacting bisphenol.

The associations between bisphenol mixtures and T1DM risk were further evaluated using the Bayesian kernel machine regression (BKMR) model (Figure 2). Figure 2A shows the overall effect of the bisphenol mixture (BPA, BPF, and BPS) on T1DM risk. An increasing trend in the estimated effect was observed as the mixture quantile increased, indicating a positive association between higher cumulative bisphenol exposure and T1DM risk. Figure 2B presents the single-exposure effects. The effect was estimated by comparing the 75th percentile with the 25th percentile, while fixing the concentrations of other bisphenols at the 25th, 50th, and 75th percentile levels. Both BPA and BPF demonstrated consistent positive associations with T1DM risk, especially when the background levels of the other bisphenols were at or above the median levels. In contrast, BPS showed weaker or no effects across the examined quantile levels (Figure 2B). The univariate exposure-response functions for each bisphenol are shown in Figure 2C. BPF had the highest posterior inclusion probability (PIP = 1.000), followed by BPS (0.992) and BPA (0.942). BPA and BPF displayed approximately monotonic increasing exposure-response patterns, whereas BPS showed a relatively flat relationship with wider credible intervals. Figure 2D shows the pairwise exposure-response functions among BPA, BPF, and BPS. The curves were largely overlapping or approximately parallel across quantiles of the other bisphenols, providing no clear visual evidence of strong pairwise interaction. Therefore, these plots were interpreted as exploratory descriptive findings rather than evidence of synergistic interaction.

### 3.5 Association of individual urinary bisphenol (adjusted by Cr) with indexes of glucose metabolism

Table 4 presents the associations between individual urinary bisphenol concentrations and glycemic traits in the adjusted models. Higher urinary BPS concentrations were associated with higher FBG (*β* = 0.987, 95% CI: 0.104–1.871, *p* = 0.029) and HbA1c levels (*β* = 0.327, 95% CI: 0.012–0.642, *p* = 0.042). No statistically significant associations were observed between BPA or BPF concentrations and FBG, HbA1c, fasting insulin, or HOMA-β after adjustment for covariates, including case–control status. Associations of ∑BPs with the glycemic traits were also not statistically significant. Crude estimates are provided in Supplementary Table S8 for descriptive comparison. Sensitivity analyses with additional adjustment for BMI, family history of diabetes, and both variables yielded generally consistent results and are presented in Supplementary Table S9.

**Table 4:** Association of individual bisphenol with FBG, HbA1C, fasting insulin and HOMA-β.

Variable	FBG	*p*	HbA1C	*p*	F insulin	*p*	HOMA-β	*p*
	*β* (95%CI)		*β* (95%CI)		*β* (95%CI)		*β* (95%CI)	
BPA	−0.399 (−1.362, 0.564)	0.413	0.197 (−0.144, 0.538)	0.254	−0.348 (−1.512, 0.816)	0.554	−8.775 (−26.962, 9.411)	0.341
BPF	−0.154 (−0.884, 0.577)	0.677	0.194 (−0.063, 0.451)	0.137	−0.361 (−1.240, 0.519)	0.418	−4.141 (−17.938, 9.656)	0.553
BPS	0.987 (0.104, 1.871)	0.029	0.327 (0.012, 0.642)	0.042	−0.830 (−1.909, 0.250)	0.130	−15.453 (−32.263, 1.393)	0.072
∑BPs	−0.186 (−1.429, 1.057)	0.767	0.394 (−0.040, 0.829)	0.075	−0.756 (−2.249, 0.738)	0.318	−16.580 (−39.860, 6.700)	0.161

Adjusted for age, sex, residence, parental education, maternal age, and vegetable consumption, case–control status.

**Figure 3:**
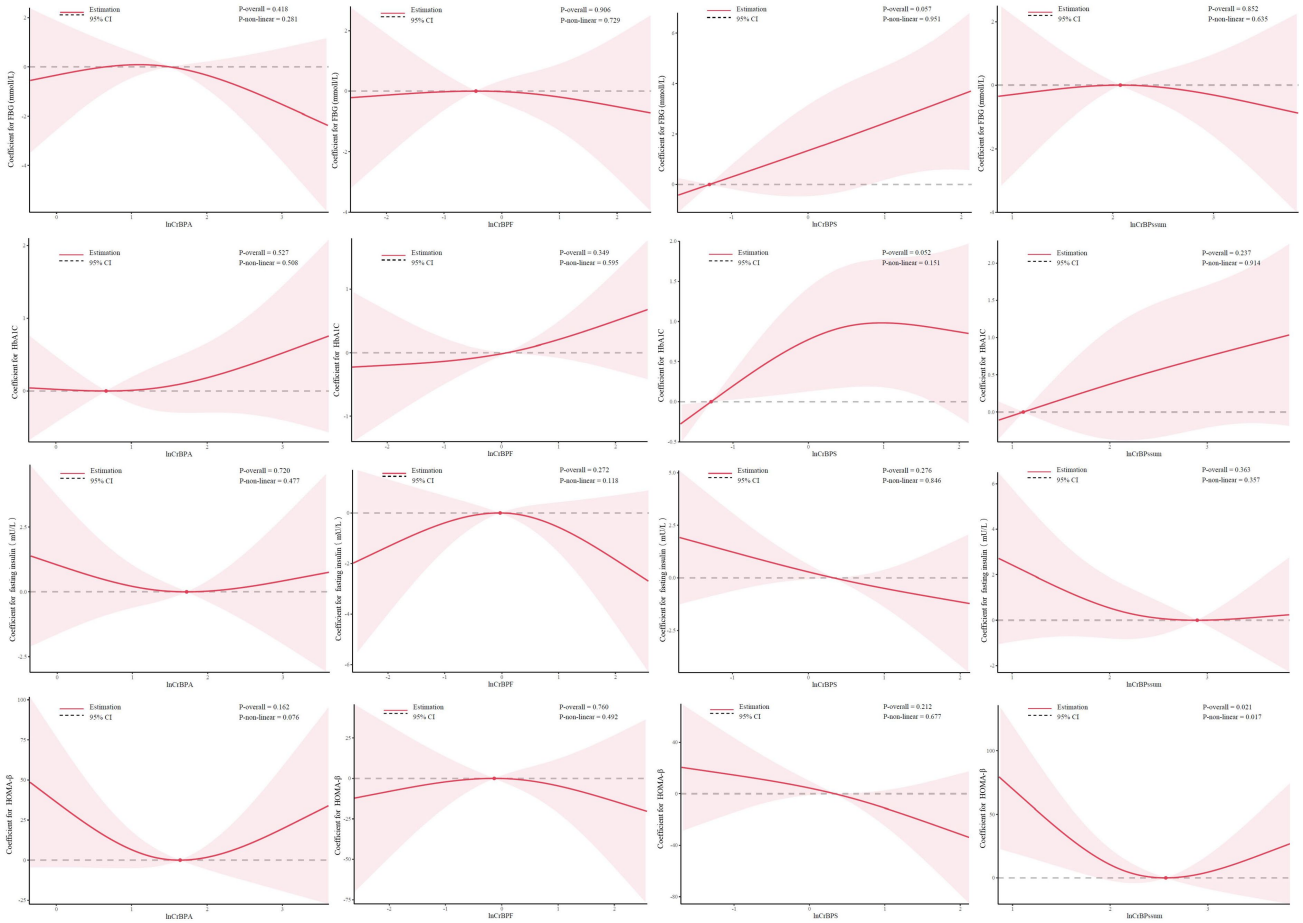
Association between bisphenol compounds and indexes of glucose metabolism by restricted cubic spline. Model adjusted for age, sex, residence, group, parental education, maternal age, and vegetable consumption, group.

Restricted cubic spline (RCS) analyses were conducted to evaluate the potential nonlinear associations between urinary bisphenol concentrations and glycemic outcomes (Figure 3). Urinary bisphenol concentrations were creatinine-adjusted and natural log-transformed prior to analysis. For FBG, no significant nonlinear relationships were detected for BPA, BPF, BPS, and BPsum in the fully adjusted models (all *p-nonlinear* > 0.05). Overall, the associations were also non-significant (all *p-overall* > 0.05), which was consistent with the linear regression results. For HbA1c, the RCS model indicated a borderline overall association for BPS (*p*-overall = 0.052), whereas no significant associations were detected for BPA, BPF and BPsum. For fasting insulin, inverse trends for BPA, BPS, and BPsum that appeared in the crude models were attenuated after adjustment. For BPF, a possible U-shaped pattern was observed in the unadjusted analyses, although the test for nonlinearity was not statistically significant (*p*-nonlinear = 0.118). For HOMA-*β*, the RCS analysis showed a significant overall association with ∑BPs (*p-overall* = 0.021) and evidence of nonlinearity (*p-nonlinear* = 0.017). The exposure–response curve suggested a potential J-shaped relationship between combined bisphenol exposure and β-cell function. No significant overall or nonlinear associations were detected for BPA, BPF, or BPS in the adjusted model.

**Figure 4:**
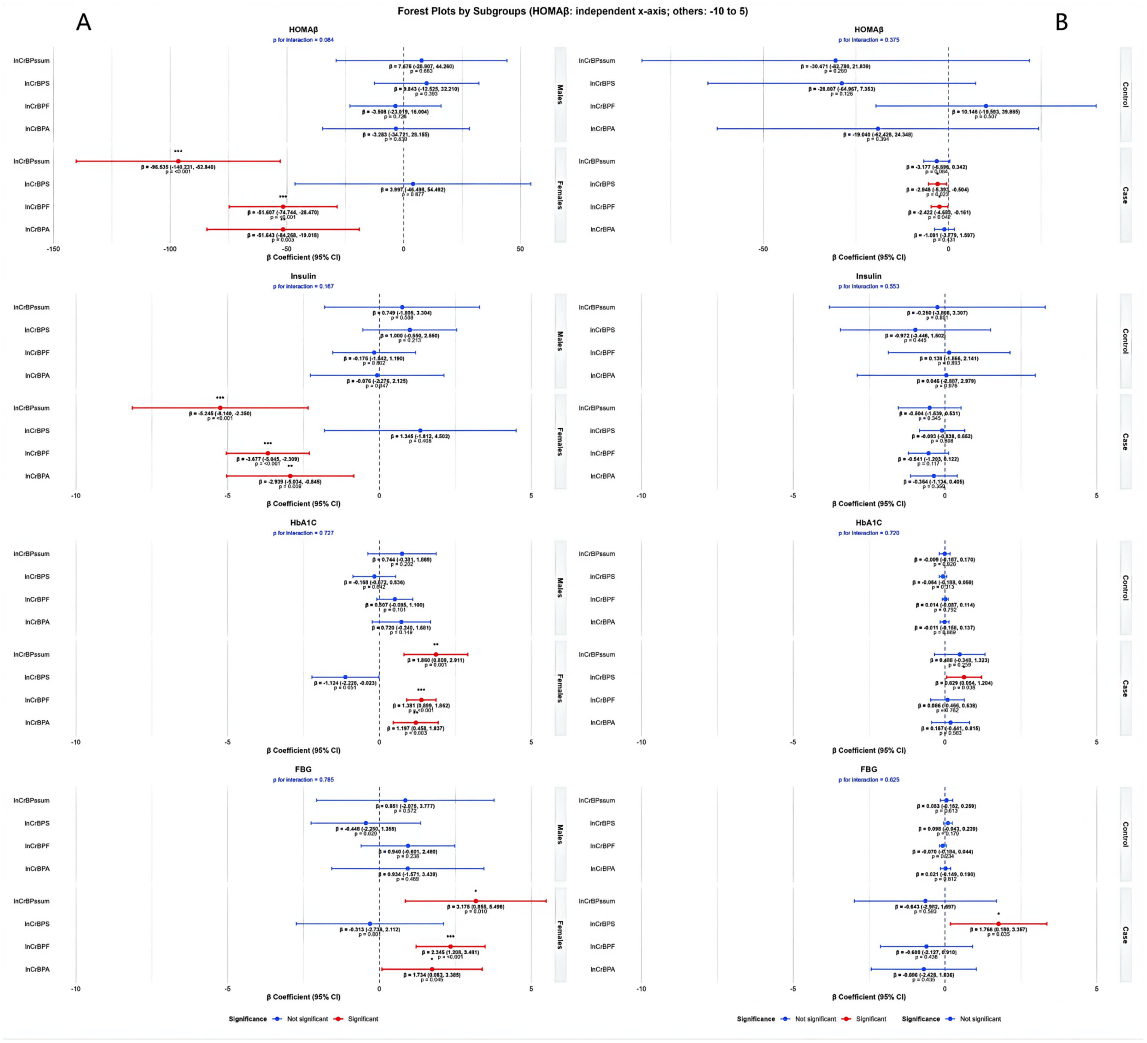
Stratified associations between urinary bisphenols and glucose homeostasis. **(A)** Sex-stratified analyses. Forest plots display regression coefficients (*β*) and 95% confidence intervals (CIs) for the associations between ln-transformed, creatinine-adjusted bisphenol concentrations and glucose-related outcomes. Associations between BPF and total bisphenols with insulin-related indices and glycemic measures appeared more evident among females, whereas fewer associations were observed among males. Models were adjusted for age, sex, residence, parental education level, maternal age, and vegetable intake. **(B)** Case–control stratified analyses. Squares represent T1DM cases and circles represent controls. Associations between BPS and glycemic traits, including HOMA-β and fasting blood glucose, were observed among cases, whereas no consistent associations were identified among controls. All estimates are presented as *β* coefficients with 95% confidence intervals (CIs), adjusted for age, sex, residence, parental education, maternal age, and vegetable intake.

We further evaluated potential effect modification by sex and case–control status using multiplicative interaction terms. Evidence of interaction by case–control status was observed for BPS in relation to FBG (*p* for interaction = 0.042) and HbA1c (*p* for interaction = 0.027). Sex-related interactions were observed for BPF in relation to fasting insulin (*p* for interaction = 0.003) and HOMA-*β* (*p* for interaction = 0.007), as well as for ∑BPs in relation to fasting insulin (*p* for interaction = 0.029) and HOMA-β (*p* for interaction = 0.004). Exploratory evidence of sex-related heterogeneity was also observed for BPF and BPS in relation to HbA1c (*p* for interaction = 0.078 and 0.093, respectively). The corresponding subgroup-specific estimates are displayed in Figure 4, and the complete interaction-test results are provided in Supplementary Table S10. Given the limited sample size, all subgroup findings were interpreted as exploratory.

## 4 Discussion

This study provides novel epidemiological evidence on the associations between urinary bisphenol exposure and both T1DM risk and glycemic regulation in children. To our knowledge, this study is among the first to examine associations of BPA substitutes (BPF and BPS), and bisphenol mixtures with T1DM and metabolic markers in children. In this case–control study conducted in northern China, we observed widespread exposure to bisphenols among children. Higher urinary concentrations of BPA and BPF, as well as bisphenol mixtures, were associated with increased odds of T1DM. In contrast, BPS was not associated with T1DM status but was related to higher fasting glucose and HbA1C levels. These patterns were more evident among girls and in children with T1DM, indicating potential differences in susceptibility across subgroups.

Compared with previous studies, urinary BPA concentrations in the present study were lower than those reported in case–control studies from Turkey (T1DM: 27.70 μg/g creatinine; control group: 25.37 μg/g creatinine) (17) and Thailand (T1DM: 31.50 μg/g creatinine; control group: 10.13 μg/g creatinine) (9), but higher than those observed in children from Egypt (T1DM: 1.8 ng/mL; control group: 1.4 ng/mL) (10). Relative to estimates reported for the general pediatric population, BPA concentrations in our study also appeared somewhat elevated. For example, a cross-sectional study of 358 Thai children reported a geometric mean BPA concentration of 1.41 ng/mL (28). Another study from Korea, based on data from the Korean National Environmental Health Survey (KoNEHS), showed a median level of 1.82 μg/L among children aged from 6–11 years (29). Additionally, two studies from China reported geometric concentrations of BPA of 0.84 μg/L and 1.17 μg/L (30, 31). By contrast, an Iranian study reported substantially higher BPA concentrations (232.60 μg/L) (32). In the present study, BPA was detected in 100% urine samples, which is consistent with exposure frequencies reported in other Chinese studies (23). Overall, these findings indicate that widespread BPA exposure among children in Shenyang and highlight the need for further investigation into potential exposure sources.

Data on urinary BPF and BPS concentrations in children with T1DM are limited. In the present study, BPF was detected in 90.91% of urine samples, whereas BPS was present in 99.09%. The geometric mean concentrations of unadjusted BPF were 1.35 μg/L in the T1DM group and 0.56 μg/L in the control group. For BPS the corresponding concentrations were 0.60 μg/L among cases and 2.12 μg/L among controls. The detection rates and concentrations of BPF and BPS observed in this study were higher than those previously reported for healthy children. For example, a study from Eastern China reported in detection rates and median concentrations of BPF at 12% and 0.19 μg/L, and BPS at 89% and 0.03 μg/L (31). Similar findings have been reported in other countries. In Thailand, BPF was present in 31% of samples with a GM of 0.013 ng/mL, while BPS was present in 16.8%, demonstrating a GM of 0.014 ng/mL (28). In the United States, BPF was identified in 66.5% of samples, and its median level was 0.35 μg/L, whereas the detection rate of BPS was 89.4%, with a median concentration of 0.37 μg/L (33). The differences in detection rates and concentrations across studies may reflect variations in study populations, exposure patterns, sample collection, and analytical procedures. Therefore, these cross-study comparisons should be interpreted with caution.

Research on the potential link between BPA exposure and T1DM has been limited and inconsistent. The initial investigation to report BPA exposure in pediatric patients was carried out by Tolga İnce et al. (17) in Turkey. It involved a total of 100 children ranging in age from 5–18 years (50 with T1DM and 50 healthy controls) (17). According to their report, there is no substantial correlation between BPA levels in urine and T1DM. However, two other case–control studies conducted in Thailand (9) and Egypt (10), both with larger sample sizes, reported higher BPA levels in children with T1DM than in controls, which is consistent with the present findings. Furthermore, our study compared BPF, BPS, and bisphenol mixture concentrations between the cases and controls. Both BPF and bisphenol mixture concentrations in the T1DM group were significantly higher compared with those in the control group, whereas BPS concentrations were found to be lower.

Moreover, based on multiple logistic regression and BKMR models, higher exposures to BPA, BPF, and bisphenol mixture were associated with increased risk of T1DM in children. To date, epidemiological evidence examining both individual bisphenols and bisphenol mixture in relation to T1DM remains limited. However, several case–control studies have reported higher urinary BPA concentrations among children with T1DM compared with controls, supporting a possible link between bisphenol exposure and diabetes risk (9, 10). In the mixture analysis, BPA and BPF appeared to contribute more strongly to the overall association with T1DM risk, whereas BPS showed weaker or null associations with disease status. Bivariate exposure-response analyses further suggested potential interactions between BPF and BPS, indicating that combined exposures may influence metabolic outcomes in a non-additive manner. These findings highlight the importance of considering real-world chemical mixtures rather than single compounds when evaluating environmental health risks.

Complementary analyses using multivariable regression further examined the associations between individual bisphenols and glycemic markers. These analyses showed that urinary BPS concentrations were associated with higher fasting glucose and HbA1c levels, whereas BPA and BPF were not independently related to glycemic markers after adjustment for potential confounders.

Experimental evidence (19) have suggested that bisphenols can interfere with endocrine processes involved in insulin secretion and glucose regulation. However, epidemiological data on this issue are still scarce and inconclusive. Most available studies have focused on type 2 diabetes in adult or gestational diabetes rather than detailed glycemic measures in children with T1DM (24, 34). Whether BPS exposure is associated with glycemic regulation in children with T1DM has not been established.

Spline analyses further indicated that the associations between bisphenol exposure and metabolic outcomes were not strictly linear. Similar patterns have been reported for endocrine-disrupting chemicals, including bisphenols, and are thought to reflect complex biological responses to low-dose exposures (35).

Subgroup analyses indicated that associations between bisphenol exposure and metabolic outcomes may vary across biological and disease contexts. Although not consistently observed across all comparisons, these patterns may reflect underlying biological differences. Evidence of interaction was observed for selected exposure–outcome pairs, including BPF and ∑BPs with insulin-related indices by sex, and BPS with glycemic traits by disease status. These patterns imply that responses to bisphenol exposure may differ depending on hormonal or metabolic background. The observations are broadly consistent with previous studies reporting sex-related differences in the associations between bisphenol exposure and markers of glucose metabolism, including findings by Stahlhut et al. (36). However, these patterns were not consistent across all analyses, and differences between subgroups were generally modest. Given the number of comparisons and the limited sample size within strata, the results should be interpreted as exploratory and require confirmation in larger studies.

Taken together, these findings suggest that bisphenol exposure may be relevant to metabolic health in children. Given the widespread presence of these compounds in food packaging and plastics, low-level exposure is likely to occur routinely in daily life. The observed relationships between bisphenol analogs, T1DM, and glycemic markers indicate a potential role in early metabolic processes and support the need for continued monitoring of BPA substitutes. Several limitations should be considered. The case–control design does not allow causal inference or determination of temporal relationships between exposure and disease onset. In addition, urinary concentrations were measured at a single time point and may not fully reflect longer-term exposure patterns, particularly given the short biological half-lives of bisphenols. The relatively small biomarker sample and single-center setting may also limit the generalizability of the findings. Further prospective studies with repeated exposure assessment, along with mechanistic investigations, are needed to better understand these relationships.

## 5 Conclusion

By examining the relationships between bisphenol exposure and metabolic profiles in children, this study expands the existing evidence regarding environmental factors related to diabetes risk. Higher urinary concentrations of BPA and BPF were associated with increased odds of T1DM, while BPS was linked to alterations in glycemic markers. With the increasing use of BPA substitutes, these findings suggest that structurally similar compounds may also be relevant to metabolic health during childhood. Future longitudinal studies and experimental models are necessary to elucidate the biological mechanisms underlying these observations.

## Data Availability

Due to ethical restrictions and participant confidentiality requirements, the raw data from this study are not publicly available. Reasonable requests for data access should be directed to the corresponding author. Requests to access the datasets should be directed lhjia@cmu.edu.cn.
