# Supplementary material for: Urinary bisphenol exposure and risk of type 1 diabetes and glycemic traits in Chinese children: a case–control study
Source: Front Nutr. 2026 Jul 29;13:1880074. doi: 10.3389/fnut.2026.1880074 (PMC13461628; doi:10.3389/fnut.2026.1880074)
Supplement: Supplementary file 1 [file Supplementary_file_1.docx]

**Supplementary Material S1**

**Sample size consideration and power**

No formal a priori sample size calculation was performed. The sample size was determined based on feasibility considerations and the availability of eligible participants during the study period. The sample size rationale was further informed by previous biomonitoring studies of environmental chemical exposure in pediatric populations.

This study enrolled 122 cases and 287 controls. A complete data subset of 110 participants (55 cases and 55 controls, aged 6–14 years) was available for bisphenol exposure and metabolic outcome analyses. Assuming a two-sided α level of 0.05, this subset provides >80% power to detect large associations (e.g., odds ratio ≈ 3.0) but limited power for modest associations (odds ratio ≈ 1.5–2.0). For continuous outcomes, the detectable standardized mean difference at 80% power was approximately 0.55–0.60 under equal variance assumptions. Accordingly, analyses involving mixture modeling and nonlinear exposure-response relationships were interpreted as exploratory (Supplementary Methods S1).

**Supplementary Methods S2**

**The assay method of urinary BPA, BPF and BPS levels**

**Preparation of Standard Solutions and Construction of Standard Curves**

The standards and corresponding internal standards were received as powders. The preparation method for The standard solutions were prepared as follows: the standard substance was placed in a 10 mL polypropylene centrifuge tube and diluted to the mark with methanol to obtain a stock solution of the standard substance. After dilution according to the concentration gradient, the final concentrations of BPA, BPF, and BPS were all 1μg/mL. The diluted solution and stock solution were stored at 4°C until further use. Dansyl chloride was dissolved in acetone to prepare a 2 mg/mL derivatization reagent, which was stored at -20°C for later use. The preparation method for The isotopically labeled internal standards were prepared as follows. The isotopically labeled internal standards 13C_12_-BPA and BPF-d10 were placed in a 10 mL polypropylene centrifuge tube and diluted to the mark with methanol to obtain a stock solution of the isotopically labeled internal standards. After dilution, according to the concentration gradient, the final concentrations of 13C_12_-BPA, BPF-D10, and 13C_12_-BPS were 1μg/mL, 1μg/mL, and 0.1μg/mL, respectively. The diluted solution and stock solution were stored at 4°C until further use. Construction of the standard curve: The amount of internal standard was 10ng. The standard curve is set with 10 concentration points, which are 0.1ng/mL, 0.2ng/mL, 0.5 ng/mL, 1.0 ng/mL, 2.0ng/mL, 6.0 ng/mL, 10 ng/mL, 15 ng/mL, 20 ng/mL, and 50ng/mL. After adding the standard solution and internal standard dilution liquid with a concentration of 1μg/mL, dry in a 40°C water bath with nitrogen blowing, add 100μl of derivatization reagent and 100μl of sodium carbonate-sodium bicarbonate buffer solution, vortex mixed, and then derivatized in a 60°C water bath for 15 min. The mixture was dried again in a 40°C water bath with nitrogen blowing, redissolve with 400μL of methanol, mixed well, and filtered into a brown vial for testing.

**Urine Sample Pretreatment**

The stored urine was thawed at room temperature, centrifuged at 4000 rpm for 10 minutes, take 1.0 mL) was collected. Then add a mixed internal standard solution of 10 μL (the concentrations of 13C_12_-BPA and BPF-d10 are both 1 μg/mL, and 13C_12_-BPS is 0.1 μg/mL), mix well, and then add 1.5 mL of 0.2M acetic acid-sodium acetate buffer solution (pH=5.2). Add 10 μL of β-glucuronidase/arylsulfatase, mix well, and hydrolyze in a 37°C water bath for 12 h. The Oasis HLB cartridge was sequentially activated with 5 mL methanol and 5 mL pure water. The hydrolyzed urine was loaded onto the cartridge, followed by washing with 5 mL pure water and 5 mL 5% methanol to remove impurities and interfering substances adsorbed onto the cartridge. The target compounds were eluted with 4 mL of methanol and the eluate was collected. Under the action of a 40°C water bath, the eluate containing the target compounds was dried with an appropriate nitrogen flow, redissolved in 400 μL of methanol, shaken and centrifuged, passed through a 0.22 μm organic filter membrane, and added to the injection vial for testing.

**Liquid Chromatography Conditions**

The separation was performed on a ZORBAX SB-C18 column (4.6 mm × 150 mm, 5 μm, Agilent, USA), and the mobile phase consisted of 0.1% aqueous formic acid (A) and methanol solution (B), with an elution program of 0-1.0 min, 70–60% (A), 1.0-5.0 min linearly reduced to 5% (A), maintained at 5% (A) from to 5-8.5 min, 8.5-9 min linearly increased to 70% (A), and 9-12 min increased to 70% (A); the flow rate was 0.6 mL/min; the column temperature was 40°C; continuous automatic injection, with an injection volume of 5μL.

**Mass Spectrometry Conditions**

Mass spectrometry analysis was performed in the positive ion electrospray ionization (ESI+) and multiple reaction monitoring (MRM) modes. The optimized parameters were as follows: gas temperature, 300°C; Gas Flow, 10 L/min; Nebulizer, 45 psi; capillary voltage, 4000 V (positive); and 3500 V (negative).

**Quantitative Method and Quality Control**

All target compounds were quantified using isotope-labeled internal standard calibration curves. 13C_12_-BPA was used as an internal standard for the calibration curve of BPA, BPF-D10 for BPF, and 13C_12_-BPS for BPS. The resulting standard curves had a correlation coefficient greater than 0.999 for all target compounds, indicating a good linear relationship within the respective concentration ranges. The recovery rates of BPA, BPF, and BPS in the sample matrix are between 92.42% and 112.32%. After every 22 samples, a midpoint calibration standard and a methanol blank were injected to monitor the drift of the instrument response and carryover contamination from the previous injection. The acceptable accuracy of the midpoint calibration standard was within the range of 85-115%. The limits of detection (LOD) were calculated based on a signal-to-noise ratio (S/N) of approximately 3, and the limits of quantification (LOQ) were calculated based on a signal-to-noise ratio (S/N) of approximately 10.

**Urine Creatinine Assay**

Urine creatinine ( Cr) was detected using the creatine oxidase method and measured with a microplate reader. Urine samples were diluted 50 times, and standard and sample wells were set up along with corresponding replicates. In the standard wells, 12 μL of standard solutions of different concentrations was added; in the sample wells, 12 μL of the sample was added to the wells. Then, 180 μL of the reagent was added to both the standard and sample wells and incubated at 37°C for 5 min. Then, 60 μL of reagent two to all wells. After incubation at 37°C for 2 min, the absorbance of each well was measured at 515 nm wavelength using a microplate reader (A1). After a further 3-minute incubation at 37°C, the absorbance of each well was measured at 515 nm using a microplate reader as A2. Urine creatinine levels were calculated based on a standard curve. The BPA concentration corrected for urine creatinine was obtained by dividing the BPA concentration by the urine creatinine concentration.

**Supplementary Methods S3**

Bayesian kernel machine regression (BKMR) was applied to evaluate the joint effects of bisphenol exposures (BPA, BPF, and BPS) on T1DM risk. BKMR analyses were implemented in R using the package “bkmr”.

Markov chain Monte Carlo (MCMC) sampling was performed with 10,000 iterations, including a burn-in period of 2,000 iterations. Posterior samples from the remaining iterations were used for inference. Default Gaussian kernel functions were used to model potentially nonlinear and non-additive relationships between exposures and outcomes.

Posterior inclusion probabilities (PIPs) were estimated to evaluate the relative importance of each exposure in the mixture. Overall mixture effects were estimated by comparing predicted outcomes when all exposures were set to different quantiles of their joint distribution relative to the median. Univariate exposure–response functions were estimated while holding other exposures at fixed percentiles.

**Supplementary Table S1.** Mass spectral parameters for the determination of bisphenols.

| Compound | RT(min) | Precursor ion | Quantitative ion | Confirm ion | Fragmentor(v) | Collision Energy | |
| --- | --- | --- | --- | --- | --- | --- | --- |
|  |  | m/z | m/z | m/Z |  |  |  |
| BPA | 10.4 | 695 | 171 | 156 | 210 | 171→54 | 161→85 |
| BPF | 9.4 | 667 | 171 | 156 | 210 | 171→50 | 156→80 |
| BPS | 8.5 | 717 | 717 | 156 | 220 | 171→50 | 156→85 |
| BPA-^13^C12 | 10.4 | 707 | 171 | 156 | 210 | 171→54 | 156→85 |
| BPF-D10 | 9.4 | 677 | 171 | 156 | 240 | 171→48 | 156→82 |
| BPS-^13^C12 | 8.5 | 729 | 171 | 156 | 220 | 171→50 | 156→85 |

Note: Abbreviations: BPA, bisphenol A; BPF, bisphenol F; BPS, bisphenol S

**Supplementary Fig.S1** Directed Acyclic Graph of association between bisphenols exposure and T1DM

**Supplementary Table S2** Multivariate models for predicting risk of T1DM

| Varialbe | OR(95%CI) | SE | P-value |
| --- | --- | --- | --- |
| Residence |  |  |  |
| Urban | Ref. |  |  |
| District | 4.244(1.636, 11.010) | 0.486 | 0.003 |
| Rural | 2.721(0.932, 7.944) | 0.547 | 0.067 |
| Mother education |  |  |  |
| Junior high school and blow | Ref. |  |  |
| Senior high school | 0.512(0.203, 1.291) | 0.472 | 0.156 |
| College and above | 0.364(0.142, 0.934) | 0.481 | 0.036 |
| Family history of diabetes |  |  |  |
| No | Ref. |  |  |
| Yes | 2.512(1.406, 4.489) | 0.296 | 0.002 |
| Intake of vegetables,times/week |  |  |  |
| <2 | Ref. |  |  |
| 2-4 | 1.033(0.567, 1.883) | 0.306 | 0.914 |
| ≥4 | 0.082(0.032, 0.212) | 0.481 | <0.001 |

**Supplementary Table S3** Characteristics of children with T1DM(cases) and healthy children(controls) tested for urine bisphenols

| Parameters | Cases(n=55) |  | Controls (n=55) | *x^2^/Z* | *P value* |
| --- | --- | --- | --- | --- | --- |
|  | n(%) |  | n(%) |  |  |
| Age(years) | 10.04±2.16 |  | 10.67±2.07 | 11.576 | 0.118 |
| Sex |  |  |  | 0.000 | 1.000 |
| Boys | 27(49.1) |  | 27(49.1) |  |  |
| Girls | 28(50.9) |  | 28(50.9) |  |  |
| BMI |  |  |  | 5.979 | 0.050 |
| Normal | 40(72.7) |  | 29(52.7) |  |  |
| Overweight | 8(14.5) |  | 9(16.4) |  |  |
| Obesity | 7(12.7) |  | 17(30.9) |  |  |
| Residence |  |  |  | 4.189（Z） | 0.131 |
| Urban | 34(61.8) |  | 42(76.4) |  |  |
| District | 4(30.9) |  | 5(55.6) |  |  |
| Rural | 17(7.3) |  | 8(32.0) |  |  |

**Supplementary Table S4** Spearman correlation coefficients of urinary BPA and its alternatives.

| Urinary bisphenols concentrations | Crude concentrations | | |  | Cr-adjusted concentrations | | |  |
| --- | --- | --- | --- | --- | --- | --- | --- | --- |
|  | BPA | BPF | BPS |  | BPA | BPF | BPS |  |
| BPA | 1 | 0.483** | -0.248** |  | 1 | 0.450** | 0.075 |  |
| BPF | - | 1 | -0.363** |  | - | 1 | -0.201* |  |
| BPS | - | - | 1 |  | - | - | 1 |  |

**Supplementary Table S5** Odds ratios and 95% confidence intervals of the associations between tertiles of urinary bisphenols (μg/g Cr ) and T1DM

|  | N | Model 1  OR(95%CI) | *P* | Model 2  OR(95%CI) | *P* |
| --- | --- | --- | --- | --- | --- |
| BPA |  |  |  |  |  |
| Q1[1.24(<2.05)] | 36 | Ref. |  | Ref. |  |
| Q2[3.80(2.05-6.15)] | 37 | 4.874 (1.71-13.91) | 0.003 | 4.869 (1.51-15.75) | 0.008 |
| Q3[14.57(≥6.15)] | 37 | 12.898 (4.22-39.34) | <0.001 | 11.033 (3.31, 36.81) | <0.001 |
| *P_trend_* |  | **<0.001** |  | **<0.001** |  |
| BPF |  |  |  |  |  |
| Q1[0.23(<0.49)] | 36 | Ref. |  | Ref. |  |
| Q2[1.29(0.49-2.88)] | 37 | 2.975 (1.08-8.23) | 0.036 | 2.600 (0.83, 8.11) | 0.100 |
| Q3[10.33(≥2.88)] | 37 | 15.000 (4.81-46.79) | <0.001 | 16.895 (4.69, 60.84) | <0.001 |
| *P_trend_* |  | **<0.001** |  | **<0.001** |  |
| BPS |  |  |  |  |  |
| Q1[0.49(<0.99)] | 37 | Ref. |  | Ref. |  |
| Q2[1.64(0.99-2.80)] | 37 | 0.385 (0.15-0.99) | 0.048 | 0.395 (0.14, 1.12) | 0.081 |
| Q3[5.07(≥2.80)] | 36 | 0.480 (0.19-1.23) | 0.126 | 0.481 (0.16, 1.44) | 0.192 |
| *P_trend_* |  | 0.287 |  | 0.361 |  |
| ∑BPs |  |  |  |  |  |
| Q1[3.96(<6.50)] | 37 | Ref. |  | Ref. |  |
| Q2[8.37(6.50-12.36)] | 36 | 2.210 (0.83-5.84) | 0.195 | 3.116 (0.97, 9.99) | 0.056 |
| Q3[27.71(≥12.37)] | 37 | 8.089 (2.84- 23.04) | <0.001 | 8.407 (2.61, 27.10) | <0.001 |
| *P_trend_* |  | **<0.001** |  | **<0.001** |  |

Note: Model 1 unajusted model. Model 2 adjusted model. Adjusted for age, sex, residence, parental education, maternal age, and vegetable intake.

**Supplementary Table S6**  Odds ratios and 95% confidence intervals of the associations between urinary bisphenols (μg/g Cr ) and T1DM

|  | Modle 3 |  |  | Modle 4 |  |
| --- | --- | --- | --- | --- | --- |
|  | OR(95%CI) | *P* |  | OR(95%CI) | *P* |
| BPA | 2.350 (1.484, 3.722) | **<0.001** |  | 2.543 (1.589, 4.069) | **<0.001** |
| BPF | 2.211 (1.533, 3.189) | **<0.001** |  | 2.324 (1.603, 3.368) | **<0.001** |
| BPS | 0.548 (0.360, 0.835 ) | **0.005** |  | 0.708 (0.487, 1.028) | **0.070** |
| ∑BPs | 2.681 (1.486, 4.837) | 0.001 |  | 3.312 (1.778, 6.170) | **<0.001** |

Note: Model 3 was djusted for age, sex, BMI, residence, parental education, maternal age, and vegetable consumption. Model 4 was adjusted for age, sex, diabetes family history, residence, parental education, maternal age, and vegetable intake.

**Supplementary Table S7** Odds ratios and 95% confidence intervals of the associations between urinary bisphenols (μg/g Cr ) and T1DM

|  | Modle 5 |  |
| --- | --- | --- |
|  | OR(95%CI) | *P* |
| BPA | 2.416 (1.493, 3.911) | **<0.001** |
| BPF | 2.383 (1.601, 3.545) | **<0.001** |
| BPS | 0.576 (0.376, 0.882) | **0.011** |
| ∑BPs | 3.064 (1.611, 5.830) | **0.001** |

Note: Model 5 was adjusted for age, sex, BMI, residence, parental education, maternal age, vegetable intake and diabetes family history.

|  | FBG |  |  | HbA1C |  | |  | | F insulin |  |  | | HOMA-β | |  |
| --- | --- | --- | --- | --- | --- | --- | --- | --- | --- | --- | --- | --- | --- | --- | --- |
|  | *β*(95%CI) | *P* |  | *β*(95%CI) | *P* | |  | | *β*(95%CI) | *P* |  | | *β*(95%CI) | | *P* |
| BPA | 1.954 (0.784, 3.124) | **0.001** |  | 1.273 (0.766, 1.779) | **<0.001** |  | | -2.530 (-4.009, -1.051) | | **0.001** |  | -40.872 (-60.831, -20.913) | | **<0.001** | |
| BPF | 1.577 (0.725, 2.428) | **<0.001** |  | 0.980 (0.612, 1.348) | **<0.001** |  | | -2.472 (-3.515, -1.429) | | **<0.001** |  | -33.073 (-47.514, -18.631) | | **<0.001** | |
| BPS | 0.054 (-1.170, 1.278) | 0.930 |  | -0.088 (-0.647, 0.472) | 0.757 |  | | 0.970 (-0.570, 2.510) | | 0.214 |  | 7.106 (-14.208, 28.419) | | 0.510 | |
| ∑BPs | 2.662 (1.110, 4.213) | **0.001** |  | 1.747 (1.079, 2.415) | **<0.001** |  | | -3.089 (-5.071, -1.107) | | **0.003** |  | -52.412 (-79.077, -25.748) | | **<0.001** | |

**Supplementary Table S8** Association of individual bisphenol with FBG, HbA1C, Fasting insulin and HOMA-β

Note: Unadjusted model.

**Supplementary Table S9** Association of individual bisphenol with FBG, HbA1C, Fasting insulin and HOMA-β

|  | FBG |  |  | HbA1C |  |  | F insulin |  |  | HOMA-β |  |
| --- | --- | --- | --- | --- | --- | --- | --- | --- | --- | --- | --- |
|  | *β*(95%CI) | *P* |  | *β*(95%CI) | *P* |  | *β*(95%CI) | *P* |  | *β*(95%CI) | *P* |
| BPA | -0.386 (-1.353, 0.581) | 0.430 |  | 0.200 (-0.146, 0.546) | 0.253 |  | -0.328 (-1.506, 0.849) | 0.581 |  | -9.282 (-27.671, 9.107) | 0.319 |
| BPF | -0.196 (-0.929, 0.537) | 0.597 |  | 0.194 (-0.066, 0.455) | 0.141 |  | -0.338(-1.228, 0.551) | 0.452 |  | -3.945 (-17.904, 10.014) | 0.576 |
| BPS | 1.179 (0.263, 2.095) | **0.012** |  | 0.365 (0.033, 0.696) | **0.031** |  | -0.856 (-1.993, 0.282) | 0.139 |  | -18.635 (-36.269, -1.000) | **0.039** |
| ∑BPs | -0.171 (-1.441, 1.099) | 0.790 |  | 0.415 (-0.032, 0.863) | 0.069 |  | -0.756 (-2.249, 0.738) | 0.318 |  | -18.461 (-42.375, 5.453) | 0.129 |

Note: Adjusted for age, sex, BMI, residence, parental education, maternal age, vegetable consumption, diabetes family history and case-control status

**Supplymentary Table S10** Interaction tests for associations between urinary bisphenol exposure and glycemic traits

| **Outcome** | **Exposure** | **Subgroup variable** | **Interaction term** | **P for interaction** |
| --- | --- | --- | --- | --- |
| FBG | lnCrBPA | Sex | lnCrBPA × sex | 0.968 |
| FBG | lnCrBPA | Group | lnCrBPA × group | 0.734 |
| FBG | lnCrBPF | Sex | lnCrBPF × sex | 0.159 |
| FBG | lnCrBPF | Group | lnCrBPF × group | 0.580 |
| FBG | lnCrBPS | Sex | lnCrBPS × sex | 0.758 |
| FBG | lnCrBPS | Group | lnCrBPS × group | **0.042** |
| FBG | lnCrBPssum | Sex | lnCrBPssum × sex | 0.515 |
| FBG | lnCrBPssum | Group | lnCrBPssum × group | 0.915 |
| HbA1c | lnCrBPA | Sex | lnCrBPA × sex | 0.938 |
| HbA1c | lnCrBPA | Group | lnCrBPA × group | 0.380 |
| HbA1c | lnCrBPF | Sex | lnCrBPF × sex | **0.078** |
| HbA1c | lnCrBPF | Group | lnCrBPF × group | 0.274 |
| HbA1c | lnCrBPS | Sex | lnCrBPS × sex | **0.093** |
| HbA1c | lnCrBPS | Group | lnCrBPS × group | **0.027** |
| HbA1c | lnCrBPssum | Sex | lnCrBPssum × sex | 0.563 |
| HbA1c | lnCrBPssum | Group | lnCrBPssum × group | 0.112 |
| Insulin | lnCrBPA | Sex | lnCrBPA × sex | 0.293 |
| Insulin | lnCrBPA | Group | lnCrBPA × group | 0.844 |
| Insulin | lnCrBPF | Sex | lnCrBPF × sex | **0.003** |
| Insulin | lnCrBPF | Group | lnCrBPF × group | 0.351 |
| Insulin | lnCrBPS | Sex | lnCrBPS × sex | 0.704 |
| Insulin | lnCrBPS | Group | lnCrBPS × group | 0.935 |
| Insulin | lnCrBPssum | Sex | lnCrBPssum × sex | **0.029** |
| Insulin | lnCrBPssum | Group | lnCrBPssum × group | 0.984 |
| HOMA-β | lnCrBPA | Sex | lnCrBPA × sex | 0.121 |
| HOMA-β | lnCrBPA | Group | lnCrBPA × group | 0.174 |
| HOMA-β | lnCrBPF | Sex | lnCrBPF × sex | **0.007** |
| HOMA-β | lnCrBPF | Group | lnCrBPF × group | 0.506 |
| HOMA-β | lnCrBPS | Sex | lnCrBPS × sex | 0.994 |
| HOMA-β | lnCrBPS | Group | lnCrBPS × group | 0.457 |
| HOMA-β | lnCrBPssum | Sex | lnCrBPssum × sex | **0.004** |
| HOMA-β | lnCrBPssum | Group | lnCrBPssum × group | 0.216 |

Note: Abbreviations: FBG, fasting blood glucose. *P* for interaction values were derived from models including an interaction term between bisphenol exposure and the subgroup variable (sex or disease status). All models were adjusted for potential confounders, including age, sex, residence, parental education, maternal age, and vegetable intake. Statistical significance was defined as *P* < 0.10.
